# Combining Transcriptomics and Proteomics to Screen Candidate Genes Related to Bovine Birth Weight

**DOI:** 10.3390/ani14182751

**Published:** 2024-09-23

**Authors:** Xiuyuan Wang, Ruili Liu, Zhenpeng Chen, Renzheng Zhang, Yanfang Mei, Xiuping Miao, Xuejin Bai, Yajuan Dong

**Affiliations:** 1 Laboratory of Animal Molecular, Shandong Black Cattle Breeding Engineering Technology Center, College of Animal Science, Qingdao Agricultural University, Qingdao 266109, China; 2 Black Cattle Seed Industry Innovation Center, Shandong Black Cattle Breeding Engineering Technology Center, College of Animal Science, Qingdao Agricultural University, Qingdao 266109, China

**Keywords:** calves, birth weight, placenta, proteomics, transcriptomics

## Abstract

**Simple Summary:**

The growth and development of fetal calves depend on nutrients from maternal circulation through the placenta, which can directly impact offspring health in early or later life. In this study, we examined how the placenta regulates calf birth weight using transcriptomic and proteomic analyses, identifying candidate genes associated with this trait. Our results indicate that a key factor affecting calf birth weight is that the placenta of high-birth-weight (HB) calves supplies more nutrients to the fetus at the transcriptomic level, characterized by enhanced nutrient transport, energy metabolism, and lipid synthesis. However, placentae from low-birth-weight (LB) calves focus more on cell proliferation and angiogenesis. At the protein level, differences in calf birth weight among Shandong Black cattle primarily arise from the energy metabolism and lipid synthesis processes while also showing significant disparities in immune function. Integrated analysis suggests that increased calf birth weight in the HB group results from efficient energy production and lipid synthesis by their placentae along with optimized cholesterol biosynthesis and metabolic pathways. Finally, we propose ELOVL5, ELOVL7, ACSL1, CYP11A1, and CYP17A1 as potential protein biomarkers that modulate the fatty acid metabolism, lipid synthesis, and cholesterol levels to influence calf birth weight.

**Abstract:**

The placenta is a vital organ in bovine reproduction, crucial for blood supply, nutrient transport, and embryonic development. It plays an essential role in the intrauterine growth of calves. However, the molecular mechanisms governing placental function in calves remain inadequately understood. Methods: We established transcriptome and proteome databases for low-birth-weight (LB) and high-birth-weight (HB) calf placentae, identifying key genes and proteins associated with birth weight through bioinformatics analyses that included functional enrichment and protein–protein interactions (PPIs). Both mRNA and protein levels were validated. Results: A total of 1494 differentially expressed genes (DEGs) and 294 differentially expressed proteins (DEPs) were identified when comparing the LB group to the HB group. Furthermore, we identified 53 genes and proteins exhibiting significant co-expression across both transcriptomic and proteomic datasets; among these, 40 were co-upregulated, 8 co-downregulated, while 5 displayed upregulation at the protein level despite downregulation at the mRNA level. Functional enrichment analyses (GO and KEGG) and protein–protein interaction (PPI) analysis indicate that, at the transcriptional level, the primary factor contributing to differences in calf birth weight is that the placenta of the high-birth-weight (HB) group provides more nutrients to the fetus, characterized by enhanced nutrient transport (*SLC2A1* and *SLC2A11*), energy metabolism (*ACSL1*, *MICALL2*, *PAG2*, *COL14A1*, and *ELOVL5*), and lipid synthesis (*ELOVL5* and *ELOVL7*). In contrast, the placenta of the low-birth-weight (LB) group prioritizes cell proliferation (*PAK1* and *ITGA3*) and angiogenesis. At the protein level, while the placentae from the HB group exhibit efficient energy production and lipid synthesis, they also demonstrate reduced immunity to various diseases such as systemic lupus erythematosus and bacterial dysentery. Conversely, the LB group placentae excel in regulating critical biological processes, including cell migration, proliferation, differentiation, apoptosis, and signal transduction; they also display higher disease immunity markers (COL6A1, TNC CD36, CD81, Igh-1a, and IGHG) compared to those of the HB group placentae. Co-expression analysis further suggests that increases in calf birth weight can be attributed to both high-efficiency energy production and lipid synthesis within the HB group placentae (ELOVL5, ELOVL7, and ACSL1), alongside cholesterol biosynthesis and metabolic pathways involving CYP11A1 and CYP17A1. Conclusion: We propose that ELOVL5, ELOVL7, ACSL1, CYP11A1, and CYP17A1 serve as potential protein biomarkers for regulating calf birth weight through the modulation of the fatty acid metabolism, lipid synthesis, and cholesterol levels.

## 1. Introduction

Beef is a great source of high-quality protein, supplying essential nutrients and offering a highly desirable eating experience [1,2]. Its elevated intramuscular fat content (IMF) enhances tenderness, juiciness, and flavor while improving texture and overall acceptability [3,4,5]. The IMF percentages in the longissimus muscle of Japanese Black, German Angus, Belgian Blue, and Holstein Friesian beef are reported to be 23.3%, 4.4%, 0.6%, and 4.7%, respectively. Consequently, Japanese Black beef demonstrates superior juiciness, marbling characteristics, and tenderness compared to other high-IMF black cattle breeds [6]. Currently, there are no native black cattle breeds in China; therefore, their breeding has become increasingly important. Birth weight is a critical trait for cattle breeding programs and serves as an influential factor affecting the production performance of beef cattle—a conclusion supported by numerous researchers [7,8,9].

Practical breeding experience indicates that lower fetal birth weight often results in diminished subsequent extrauterine growth, particularly noted in beef cattle [10]. Previous studies have established a robust association between birth weight and adult weight, with the former promoting subsequent muscle development [11,12,13]. Furthermore, it has been demonstrated that the intrauterine environment is a critical determinant of birth weight, influenced by factors such as placental function, hormone levels, and metabolic capacity [14]. Correspondingly, intrauterine growth restriction induces long-term alterations in organ development [15,16]. The placenta operates as a dynamic organ integral to fetal intrauterine development through various mechanisms, including gas exchange and nutrient transport [17]. Changes in the placental proteome significantly affect fetal growth and are closely associated with both birth weight and functional effector gene expression [18,19,20]. Genes expressed extensively or specifically within the placenta also regulate its development while influencing fetal growth. For instance, the placenta-expressed transcript 1 (PLET1) protein redistributes from the cytoplasm to the apical side of trophoblast cells as gestation progresses [21], playing a pivotal role in establishing a stable trophoblast–endometrial epithelial layer that is essential for maintaining fetal growth [21]. Glial cells missing-1 (Gcm1) promote chorioallantoic branching during placental morphogenesis throughout fetal development [22,23]. Additionally, placenta-specific protein 1 (Plac1) facilitates fetal growth by regulating trophoblast invasion and migration [24]. These findings indicate that numerous genes are activated within both placental and decidual tissues affecting trophoblast attachment and outgrowth; this can even lead to early embryonic lethality. However, there remains limited research on how placental factors influence bovine fetal development at the molecular level. Therefore, investigating this topic holds considerable scientific significance.

We selected Shandong black cattle as our experimental model, representing the first new germplasm of beef cattle successfully cultivated through somatic cell nuclear transfer in China. This breed is characterized by its fresh and tender meat, which possesses a desirable flavor and a distinctive marbling pattern, often referred to as ‘snowflake meat’. It is noted for its delicacy, juiciness, and high protein content. Compared to pork, its amino acid composition aligns more closely with human nutritional requirements and can enhance the body’s disease resistance [25]. High-throughput sequencing has been extensively employed to investigate placental transcription and protein levels [26], revealing numerous placental proteins that significantly influence fetal characteristics in various species, including pigs [27], yaks [28], and horses [29], and traits like litter size in pigs [30]. Our objective was to identify the placental protein markers associated with intrauterine calf development through proteomic and transcriptomic analyses of low- and high-weight calves. We screened specifically expressed placental proteins relevant to their intrauterine development, conducted functional enrichment analyses along with protein–protein interaction assessments, and validated key identified proteins using qRT-PCR and Western blot techniques. Our findings elucidate the critical biological processes and molecular mechanisms underlying fetal development in utero. Furthermore, they provide essential foundational knowledge for future investigations into the effects of the placenta on fetal development within the uterus, ultimately contributing to advancements in cattle breeding.

## 2. Materials and Methods

### 2.1. Animals and Placenta Sample Collection

The Shandong black cattle (two years old) were selected from Zhaofu Agriculture Co., Ltd. (Shandong, China), fed the same diets, and provided unrestricted access to drinking water during pregnancy. After delivery, we measured the body shape of 20 newborns, including the birth weight, average thigh circumference (the cattle were made to stand and the widest part of the thigh was measured), average chest circumference (the circumference of a circle around the chest perpendicular to the body axis from the posterior angle of the scapula), average body height (measuring the distance vertically from the highest point of the cattle’s shoulder (withers) to the ground), and daily weight gain (continuous measurement for one month). In order to investigate the molecular mechanism of the placenta and calf birth weight, we divided the placenta into two groups based on the calf birth weight (LB group: a birth weight of 28.942–30.128 kg; HB group: a birth weight of 33.983–34.667 kg), namely, the low-weight-calves group (LB) and the heavy-weight-calves group (HB), and conducted transcriptomic and proteomic library construction, respectively. Each group consisted of 3 experimental samples.

### 2.2. RNA Extraction, Library Construction, and Sequencing

The TRIzol Reagent kit (G-CLONE, Beijing, China) was utilized to extract the total RNA of the placental tissue, following the instructions. The purity and concentration of the total RNA were determined by the Nano Drop 2000 ultramicrospectrophotometer (Thermo, Waltham, MA, USA), and the integrity of the RNA was detected by 1% agarose gel electrophoresis. Subsequently, the eukaryotic mRNA was enriched and divided into short fragments, and then finally transcribed into cDNA. Libraries were created using the TruSeq RNA Library Prep Kit v2 (Illumina, San Diego, CA, USA), and fragment sizes were verified with the 2100 Bioanalyzer (Agilent, Santa Clara, CA, USA). Libraries were sequenced using the HiSeq 4000 System (Illumina, San Diego, CA, USA) at the CHUSJ.

### 2.3. Quality Control, Annotation, and Differential Expression Analysis

Reads obtained from the sequencing machines included raw reads containing adapters or low-quality bases. Therefore, to obtain high-quality clean reads, we used FastQC_v0.11.8 software to evaluate the quality of the sequencing data obtained from the machine, including the base quality, GC content, PCR replicates, etc. [31]. We used Trimomatic_v0.39 software to filter adapter sequences, primer sequences, and low-quality sequencing data. We set the average base mass to 25 and the minimum read lengths to 50 bp, while maintaining the output as paired reads [32]. We constructed an index file of a bovine reference genome (Ensembl_release106) using the genome alignment software STAR (v2.5.3. a) [33]. Then, we established a reference genome index and used HISAT2_v2.2.1 to locate the contralateral clean reads to the reference genome. Parameters, such as 2.4 [34], were set to the default values. The mapping readings for each sample were assembled using StringTie v1.3.1 [35,36]. For each transcription region, we used RNA-Seq by Expectation Maximization (RSEM-1.3.0) [37] software to calculate the FPKM (per kilobase per million mapped reads of transcription fragment) values to quantify their expression abundance and changes. The correlation coefficients between two copies were then calculated to evaluate the repeatability between the samples. Principal component analysis (PCA) was used with gmodels (http://www.rproject.org/, accessed on 20 November 2022). Principal component analysis is mainly used to reveal the structure/relationships of samples/data. Differential gene expression analysis was performed on the ssRNA-Seq data using DESeq2_v3.19 [38], with a set threshold of a fold change ≥ 1.5 and a *p*-value < 0.05 for the DEGs.

### 2.4. Protein Extraction and Digestion

The concentration of superalbumin was determined by the BCA protein detection kit, and then 100 μg of protein per group was diverted to the Eppendorf tube (eventual volume: 100 μL) and 2 μL of 0.5 M TCEP was added. The placental protein was hatched (1 h, 37 °C) and 4 μL of 1 M iodoacetamide was enrolled. The incubation, protected from light, lasted for 40 min. Subsequently, five volumes of −20 °C acetone were utilized to precipitate the proteins (−20 °C, one night). An amount of 1 mL acetone aqueous solution was utilized to wash the precipitates, and then redissolved to 100 μL (added 100 mM TEAB). The sequence-level modified trypsin was added to digest the proteins (37 °C, one night). Thereafter, the placental peptide mixture was desalted, lyophilized, and quantified.

### 2.5. iTRAQ Labeling and HPLC Fractionation

The resultant peptide mixture was labeled with the iTRAQ-8Plex Isobaric Mass Tag Labeling Kit (PreOmics, Martinsried, Germany) following the manufacturer’s instruction. The labeled peptide samples were then pooled and lyophilized in a vacuum concentrator. The peptide mixture was redissolved in buffer A (20 mM ammonium formate in water at a pH of 10.0) and subjected to high-pH separation using the Ultimate 3000 system (Thermo Scientific, Waltham, MA, USA) connected to a reverse-phase column (XBridge C18 column, Thermo Fisher Scientific, Waltham, MA, USA). The high-pH separation was conducted utilizing a linear gradient from 5% B to 45% B over a duration of 40 min (where B consisted of 20 mM ammonium formate and 80% acetonitrile at a pH of 10.0). Under the initial conditions, the column was re-balanced for 15 min, the column flow rate was 1 mL/min, and the column temperature was 30 °C. Then, 12 scores were collected, and each fraction was dried for the next step.

### 2.6. Liquid Chromatography Tandem–Mass Spectrometry(LC-MS/MS) and Data Analysis

The peptide was redissolved in 0.1% formic acid aqueous solution. The analysis was performed using the Orbitrap Fusion™Lumos™Tribrid™ online nanospray LC-MS/MS connected to the EASY-nLC 1200 system (Thermo Fisher Scientific, Waltham, MA, USA). A 4 μL peptide sample was introduced onto both the trap column and the analytical column. Separation was performed using a 90-min gradient, transitioning from 5% to 32% B (where B consisted of 0.1% formic acid in acetonitrile). The flow rate through the column was maintained at 600 nL/min, with the column temperature set at 40 °C. An electrospray voltage of 2 kV was applied at the inlet of the mass spectrometer. The parameters were as follows: (1) MS: scan range (*m*/*z*) = 350–1550; resolution = 60,000; AGC target = 4 × 10^5^; maximum injection time = 50 ms; included charge states = 2–6; dynamic exclusion = 30 s; (2) HCD-MS/MS: resolution = 30,000; isolation window = 1.2; AGC target = 7 × 10^4^; the maximum tandem mass spectra were processed by Robinson’s method [39]. The peak DB was established, taking trypsin as the digestive enzyme, by searching the xxxx database (xxx entries). The fragment ion mass tolerance of the peak DB was 0.02 Da and the parent ion tolerance was 10 ppm. The peptides were filtered with 1% FDR and 1 unique. The M injection time = 120 ms; collision energy = 38. The reference criteria for screening differentially expressed proteins of the placenta were a fold change ≥ 1.5 and *p*-value < 0.05.

### 2.7. Bioinformatics Analyses

In this study, the R-package clusterProfiler(4.0) was used for the GO and KEGG enrichment analyses [40,41,42]. GO terms could be divided into the following three categories: biological processes (BPs), cellular components (CCs), and molecular functions (MFs). An FDR < 0.05 indicates significant enrichment. STRING (http://string.embl.de/, accessed on 17 March 2023) and Cytoscape were applied to construct the PPI network [43]. The co-expressed DEGs/DEPs were used in STRING_v11.5 and Cytoscape_v3.9.1.

### 2.8. Quantitative Real-Time PCR

According to the sequencing results, we selected 8 upregulated and 8 downregulated genes for validation, respectively, and the qrt-PCR validation was performed in the same placenta tissue that was used for the sequencing (3 LB placenta sample and 3 HB placenta sample). TransZol was applied for the RNA extraction and the cDNA was generated utilizing the reverse transcriptase kit. Thereafter, primers (Table S1) were designed (Primer premier 5) and the qRT-PCR method was applied (SYBR premix Ex Taq™ and LightCycler 480) following the undermentioned procedure: 3 min (95 °C), 40 cycles for 15 s (95 °C), annealing temperature (15 s), and 20 s (72 °C). The relative mRNA level was computed (2^−ΔΔCT^ method) and all of the analyses were repeated in triplicate. The mRNA expression results were processed utilizing SPSS version 20.0.

### 2.9. Western Blot

The placenta protein lysates were produced (RIPA lysis buffer) and a BCA kit was utilized to determine and then separate the protein concentration (SDS-PAGE). Thereafter, the placental protein was diverted to a PVDF membrane after electrophoresis and blocked (skimmed milk). Subsequently, the antibodies for the GAPDH and differential proteins were applied for immunoblotting. The GAPDH (1:1000, item No.AC001), purchased from ABclonal Technology Co. Ltd. (Wuhan, China), was selected as the primary antibody. The secondary antibody, also purchased from ABclonal Technology Co. Ltd., was goat anti-rabbit IgG (H + L) (1:2000). A luminescence kit was utilized to expose the proteins, and the film was analyzed (ImageJ 1.39u) to compute the expression level. The candidate placental protein marker P21-activated kinase 1 (PAK1) (1:1000, item No. A25545) and Integrin α3β1 (ITGA3) (1:1000, item No. A17502), purchased from ABclonal Technology Co. Ltd., were compared with the GAPDH expression using Image J_v1.8.4.

### 2.10. Statistical Analysis

This study used SPSS 20.0 to process the data and expressed them as mean ± standard deviation (SD). The data were analyzed using a 1-way analysis of variance with Dunnett’s multiple comparisons, Sidak’s multiple comparisons, or Fisher’s exact test, as appropriate, and unpaired *t* tests and simple linear regressions using GraphPad Prism, version 9.1.2 (GraphPad Software, San Diego, CA, USA) [44]. Probability values < 0.05 were considered statistically significant.

## 3. Results

### 3.1. Characterization of Calves

**Figure 1:**
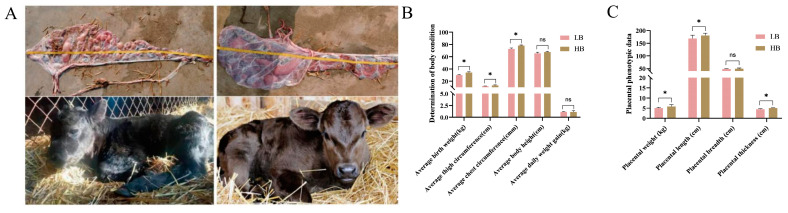
Phenotypes of the placenta and fetus. (**A**) Placenta and neonates from the LB group (left) and HB group (right). (**B**) Bar chart of the neonate phenotype data. (**C**) Statistical bar chart of the placental phenotype data. A *t* test was used, with * indicating *p* < 0.05 and ns indicating no statistically significant difference. The birth weight, thigh circumference, and chest circumference of the HB litter were significantly higher than those of the LB litter (*p* < 0.05). This suggests that the HB neonates have greater intrauterine development. The placental weight, length, and thickness in the HB group were significantly higher than those in the LB group (*p* < 0.05), but there was no significant difference in the placental width.

After delivery, we immediately measured the phenotype data of the calves and placenta in the Shandong black cattle (Figure 1A). Based on the data of 20 calves, we found significant differences (*p* < 0.05) in their weight. They were divided into two groups based on the weight, namely, the LB group, with an average birth weight of 29.535 ± 0.593 kg, and the HB group, with an average birth weight of 34.325 ± 0.342 kg. Interestingly, compared with the LB group, the thigh and chest circumference of the calves in the HB group were significantly increased (*p* < 0.05) (Figure 1B). At the same time, there were differences in the placental weight, length, width, and thickness between the LB group and the HB group (*p* < 0.05). Specifically, the weight, length, and, compared with LB group, placental thickness in the HB group was significantly increased (*p* < 0.05). However, there was no significant difference in the placental width (Figure 1C).

### 3.2. Overall Transcriptomic and Proteomic Analysis Statistics

**Figure 2:**
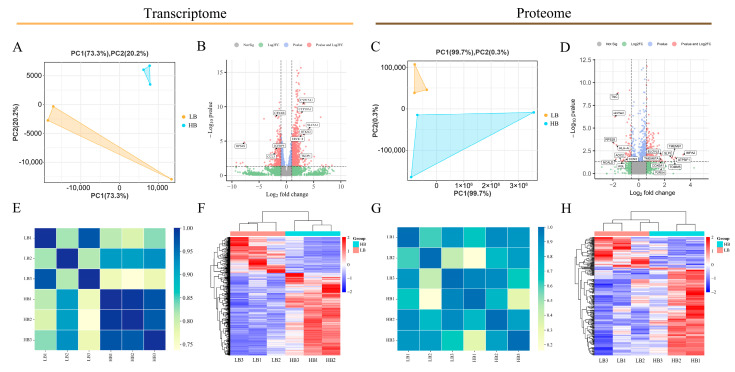
Comparison of the DEGs and DEPs between the LB and HB calves. (**A**) Principal component analysis of all quantitative RNA. (**B**) DEGs volcano map. (**C**) Principal component analysis of all quantitative protein. (**D**) DEPs volcano map. (**E**) Intra-transcriptome correlation heat map. (**F**) DEGs heat map. (**G**) Intra-proteome correlation heat map. (**H**) DEPs heat map. In (**B**,**D**), a red dot indicates that the *p*-value and log2(FC) of the DEGs/DEPs reached the threshold, a green dot indicates that the log2(FC) of the DEGs/DEPs reached the threshold, and a blue dot indicates that the *p*-value of the DEGs/DEPs reached the threshold. Gray dots indicate insignificant differences. A total of 1494 DEGs and 294 DEPs were detected. There were 1036 upregulated genes and 217 upregulated proteins. There were 458 downregulated genes and 77 downregulated proteins. The *x*-axis represents the log2(fold change) and the *y*-axis represents the −log10 (*p*-value). The dashed line represents the threshold (fold change > 1.5, *p* < 0.05), plotted according to its log2(fold change) and −log10 (*p*-value). For (**E**,**G**), each column and row in the graph represents a sample; the bluer the color, the higher the correlation; the yellower the color, the lower the correlation. For (**F**,**H**), each column in the figure represents a sample, and each row represents a gene. The expression levels of the genes in the different samples are indicated by different colors. The redder the color, the higher the expression level, and the greener the color, the lower the expression level.

We obtained 41,656,056 (on average) and 40,878,786 (on average) high-quality clean reads from the LB and HB calves, respectively. The mapping ratio of clean reads to the bovine genome was 99.09% and 99.17%. The Q20, Q30, and GC contents ranged from 95.97 to 96.66%, 89.81 to 91.59%, and 45.98 to 48.88%, respectively (Table S2). Principal component analysis (PCA) and Pearson’s correlation coefficient were utilized to assess the sample reproducibility. The PCA plot (Figure 2A) of the transcriptome data for the newborn calf placenta samples indicated that the replicates of low-born recombinant placenta samples clustered in the lower-left corner, with two being more tightly clustered, while one was slightly dispersed; conversely, high-born recombinant placenta repeat samples clustered in the upper-right corner, with higher clustering. In contrast, for the proteomics data (Figure 2B), low-born recombinant placenta repeat samples exhibited higher clustering in the upper-left corner, while high-born recombinant placenta repeat samples were more dispersed in the lower-left corner. The heat map of the transcriptome clustering revealed that both groups of placenta samples did not cluster together (Figure 2C), showing a strong genetic correlation between genes with obvious clustering patterns (Figure 2D). Consistently, sample clustering results (Figure 2E) as well as each protein’s performance (Figure 2F) in the proteomics data aligned with previous observations. The findings from the PCA diagrams and heat maps indicate the good reproducibility of the placental samples in this study despite the significant inter-group differences, enabling the subsequent screening and analysis of genes related to the initial birth weight in the newborn calves. In the transcriptome data, we identified a total of 22,358 genes, including 1494 DEGs, with 458 downregulated DEGs and 1036 upregulated DEGs (Figure 2B). Among them, *RPS4X*, *SLC5A1*, *RTKN2*, *CYP17A1*, *CYP19A1*, and *TKDP1* exhibited particularly large differential expression ratios. Through mass spectrometry analysis, we detected a total of 29,637 peptides and identified 3.844 proteins. We assessed the distribution of peptide numbers and annotated protein numbers in different databases (Figure S1A,B). Additionally, the specific distribution information of peptides is presented in Figure S1. Furthermore, we discovered 294 DEPs in different placenta groups. In the HB calves, these comprised 217 upregulated proteins and 77 downregulated proteins (Figure 2D). Specifically, IMPA2, ATP5IFl, TMEM97, LPI, and TOMM5 exhibited particularly large differential expression ratios.

### 3.3. Functional Analysis of DEGs

**Figure 3:**
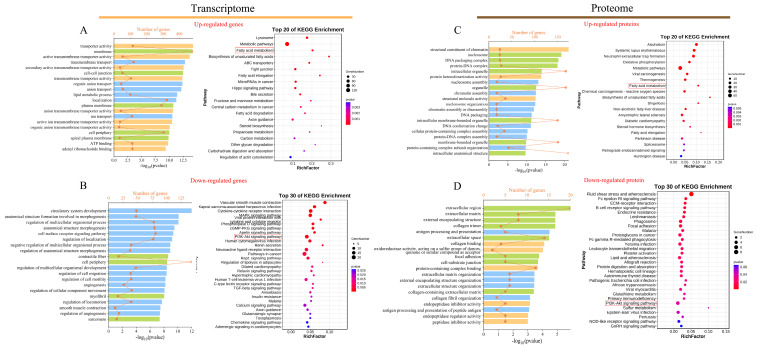
GO and KEGG pathways are enriched. (**A**,**B**) GO (left) and KEGG (right) enrichment analysis of up- and downregulated DEGs. (**C**,**D**) GO (left) and KEGG enrichment analysis of up- and downregulated DEPs (right). For the GO enrichment analysis diagram, the *x*-axis represents the gene count and the −log10 (*p*-value); the *y*-axis represents the names of the gene enrichment GO classes. Blue represents the biological process category, orange represents the cell component category, and green represents the molecular function category. For the KEGG enrichment analysis diagram, the *y*-axis represents the name of the KEGG class for the pathways, and the *x*-axis represents the Rich factor. The size of the dots corresponds to the count, as shown in the legend.

Upregulated and downregulated DEGs were employed for the functional enrichment analysis in the Blast2GO and KEGG databases, respectively. In the transcriptomic comparison between the LB and HB calves, upregulated DEGs enriched 1482 significant Gene Ontology (GO) terms (*p* < 0.05) (Figure 3A). Specifically, with respect to the molecular function, the transporter activity, active transmembrane transporter activity, secondary active transmembrane transporter activity, and general transmembrane transporter activity were significantly enriched. Notable enrichments in biological processes included transmembrane transport, organic anion transport, and lipid metabolic processes. Regarding the cellular components, upregulated differential genes demonstrated significant enrichment, primarily at the membrane structures and cell–cell junctions. The enrichment results of the downregulated DEGs (Figure 3B) exhibited marked differences from those of the upregulated DEGs; a total of 1426 GO terms were enriched within this category. These encompassed circulatory system development, anatomical structure formation involved in morphogenesis, the regulation of biological processes pertinent to multicellular organismal functions, as well as cellular components such as contractile fibers and cell peripheries. The KEGG enrichment analyses revealed substantial disparities between upregulated and downregulated DEGs concerning both the quantity and types of pathways identified. Specifically, the 58 pathways exhibiting significant enrichment among upregulated DEGs (Figure 3A) predominantly included lysosomal functions, metabolic pathways, the fatty acid metabolism, the biosynthesis of unsaturated fatty acids, ABC transporters, along with fatty acid elongation pathways. Conversely, the 39 pathways demonstrating significant enrichment among downregulated DEGs (Figure 3B) were primarily associated with vascular smooth muscle contraction, Kaposi sarcoma-associated herpesvirus infection, cytokine–cytokine receptor interactions, MAPK signaling pathway activities, PI3K-Akt signaling pathway dynamics, alongside regulatory mechanisms governing lipolysis in adipocytes.

### 3.4. Functional Analysis of DEPs

The DEPs underwent functional enrichment analysis utilizing both the Blast2go and KOBAS databases at the protein level. A total of 671 significant GO terms were enriched for the upregulated DEPs (Figure 3C). In terms of cellular components, nucleosomes and DNA packaging complexes demonstrated significant enrichment. Concerning molecular functions, structural constituents of chromatin, protein heterodimerization activity, and intracellular organelle functions were prominently enriched. Additionally, nutrient-related processes, such as the very-long-chain fatty acid biosynthetic process, fatty acid elongation, the fatty acid metabolic process, and the lipid biosynthetic process, were significantly represented within the biological processes. Furthermore, 73 notable pathways were identified among the upregulated DEPs in the comparison between the LB and HB cows (Figure 3C), including alcoholism and systemic lupus erythematosus. Among these significantly enriched pathways are those associated with bovine development such as metabolic pathways, amyotrophic lateral sclerosis, and fatty acid biosynthesis. Conversely, the downregulated DEPs exhibited enrichment for 439 significant GO terms (Figure 3D). Specifically, they encompassed biological processes including antigen processing and presentation, extracellular matrix organization, and external encapsulating structure organization, alongside cellular components such as the extracellular region and extracellular matrix. Subsequently, these downregulated proteins were found to be involved in 28 pathways primarily related to fluid shear stress and atherosclerosis, the Fc epsilon RI signaling pathway, and ECM–receptor interaction.

### 3.5. Integrative Analysis of Proteomics and Transcriptomics

**Figure 4:**
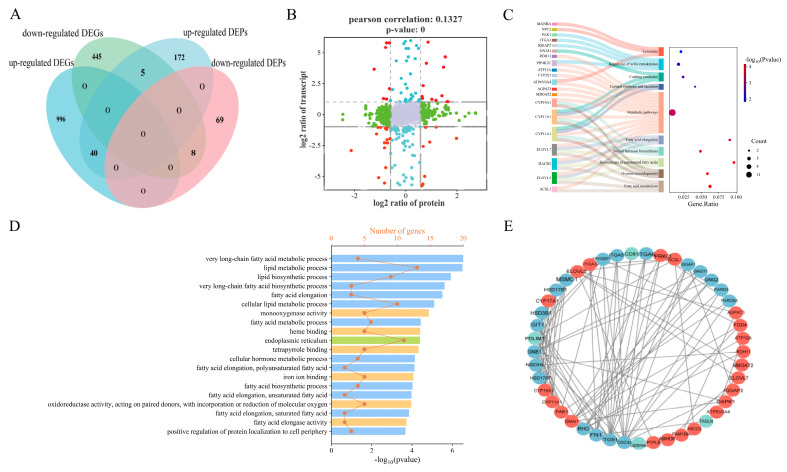
Correlation analysis between the proteomics and transcriptomics data. (**A**) The Venn diagram of the DEGs/DEPs. Pink represents the downregulated DEPs, lighter blue represents the upregulated DEPs, darker blue represents the upregulated DEGs, and light green represents the downregulated DEGs. A total of 53 pairs of DEGs/DEPs displayed discrepancies in the LB vs. HB fetal calf comparison (mRNA and protein levels), which were classified into 40 upregulated and 8 downregulated. (**B**) Nine quadrant diagrams. Different points represent genes/proteins with different expression trends. Gray indicates the non-overlapping genes/proteins. Red represents the significant differences in protein and gene expression, green represents the differences in protein expression, and blue represents the differences in transcriptional expression. (**C**) The significant pathways of co-expressed DEGs/DEPs. The *y*-axis represents the names of the gene-enriched KEGG categories, and the *x*-axis represents the Rich factor. The genes in the pathway are shown on the left. (**D**) The GO enrichment analysis of the co-expressed DEGs/DEPs. The *x*-axis represents gene count and −log10 (*p*-value); the *y*-axis represents the names of the gene enrichment GO classes. (**E**) Predicted regulatory PPI networks based on the shared co-expressed DEGs/DEPs with the same trend and their potential targeted genes. The confidence level is 0.7. The red exhibits the upregulated DEGs/DEPs and the blue exhibits the downregulated DEGs/DEPs.

To further investigate the core GO terms and pathways associated with the LB and HB cattle, a comprehensive analysis of DEGs/DEPs was performed to identify corresponding gene products and proteins. The overlapping components are illustrated in the Venn diagram (Figure 4A), revealing that 1194 out of 1494 DEGs lack corresponding coding proteins in the protein database. Concurrently, 115 coding proteins for these DEGs exhibited no significant expression differences in the proteomic data (*p* > 0.05). Similarly, among the 155 DEPs’ corresponding genes, no significant differences were observed in the transcriptome database (*p* > 0.05). In contrast, 53 DEGs, along with their respective coding proteins, demonstrated differential expression at both the transcriptomic and proteomic levels; these were categorized into 40 upregulated and 8 downregulated entities. Notably, only five DEGs/DEPs were found to be downregulated at the transcriptome level while being upregulated at the proteome level; detailed information is provided in Figure 4A. Furthermore, results from the nine-quadrant diagram (Figure 4B) indicated a low Pearson correlation coefficient (r = 0.1327), with protein quadrants exhibiting an expression pattern consistent with their transcriptomic counterparts. Subsequently, we conducted GO and KEGG analyses on identified DEGs/DEPs within both proteomics and transcriptomics datasets, which revealed ten pathways related to cattle growth (Figure 4C), including the fatty acid metabolism, ovarian steroidogenesis, and biosynthesis of unsaturated fatty acids. Besides fatty acid elongation, cortisol synthesis and secretion also showed enrichment. Additionally, the GO analysis aimed to elucidate the biological processes pertinent to cattle growth. Ultimately, the metabolic processes associated with very-long-chain fatty acids, the lipid metabolism, and lipid biosynthesis were significantly enriched, alongside monooxygenase activity and heme binding within molecular functions (Figure 4D).

### 3.6. Protein–Protein Interaction Analysis

In the PPI network (Figure 4E), the 45 co-expressed differential genes/proteins identified were preserved, as illustrated in Figure 4D. Among these, ELOVL5 emerged as a highly interconnected upregulated protein, followed by ACSL1, GNAI1, and AGPAT3, which exhibited robust interactions with proteins such as ACSL1, AGPAT3, MBOAT2, and PTPLB. Notably, this study revealed that only three differentially expressed genes/proteins (DEGs/DEPs)—PDLIM1, CD81, and SLC23A2—were significantly downregulated within the PPI network; among them, PDLIM1 displayed a greater number of interaction connections.

### 3.7. qRT-PCR and Western Blot Validation of Candidate DEGs/DEPs

**Figure 5:**
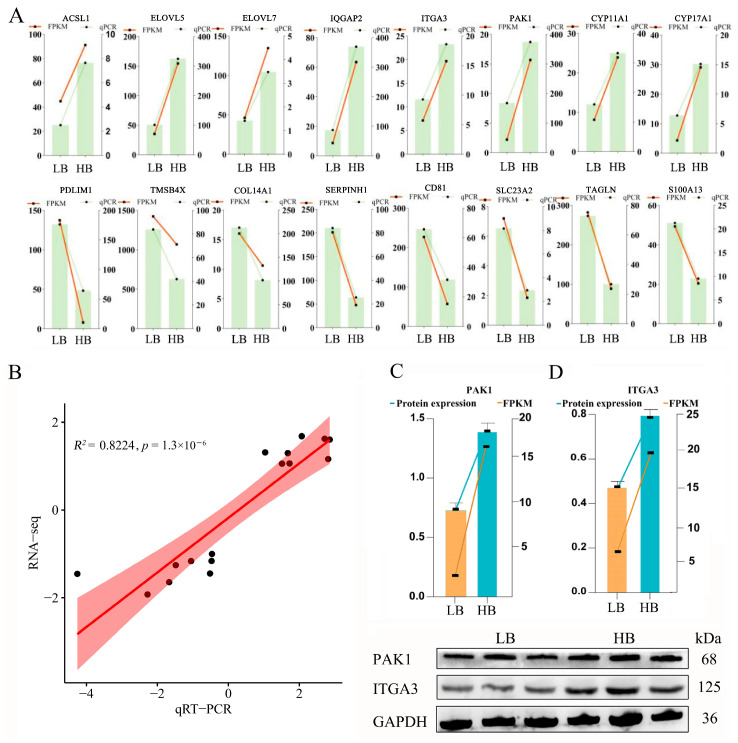
Data validation through qRT-PCR and Western blot. (**A**) Comparison of the 16 mRNAs using qRT-PCR and RNA-Seq, where the orange reflects the qRT-PCR outcomes and the blue indicates the RNA-Seq outcomes. The *x*-axis represents the names of the genes, and the *y*-axis represents the log2(FC). (**B**–**D**) The Western blot results analysis of PAK1 and ITGA3 in different groups. Values are expressed as means ± SEM (*n* = 3). The left *y*-axis is the protein expression, the right *y*-axis is the FPKM value, and the *x*-axis is the grouping. *ACSL1*, acyl-CoA synthetase long-chain family member 1; *ELOVL5*, ELOVL fatty acid elongase 5; *ELOVL7*, ELOVL fatty acid elongase 7; *IQGAP2*, IQ motif containing GTPase-activating protein 2; *ITGA3*, integrin subunit alpha 3; *PAK1*, p21 (RAC1)-activated kinase 1; *CYP11A1*, cytochrome P450 family 11 subfamily A member 1; *CYP17A1*, cytochrome P450 family 17 subfamily A member 1; *PDLIM1*, PDZ and LIM domain 1; *TMAB4X*, thymosin beta 4 X-linked; *COL14A1*, collagen type XIV alpha 1 chain; *SERPINH1*, serpin family H member 1; *CD81*, CD81 molecule; *SLC23A2*, solute carrier family 23 member 2; *TAGLN*, transgelin; *S100A13*, S100 calcium-binding protein A13.

To verify the expression levels of these pairs of genes and to acquire a deeper understanding of intrauterine growth mechanisms, we selected 16 potential candidate DEGs/DEPs with the same trend in the LB and HB calves. These included the following: *ACSL1*, *ELOVL5*, *ELOVL7*, *IQGAP2*, *ITGA3*, *PAK1*, *CYP11A1*, *CYP17A1*, *PDLIM1*, *TMSB4X*, *COL14A1*, *SERPINH1*, *CD81*, *SLC23A2*, *TAGLN*, and *S100A13*. The expression trend of these chosen genes in the qPCR analysis displayed a consistent trend, as in the RNA-Seq analysis (Figure 5A). The coefficient of determination (R^2^) was 0.8224 (Figure 5B), indicating a robust fit, and the sequencing data are appropriate for subsequent research analyses. Simultaneously, we utilized Western blotting to validate the expression of Hub DEPs in the placenta, employing GAPDH as a reference protein. PAK1 and ITGA3 were chosen as candidate proteins due to their significant biological functions. The protein expression results were consistent with those from the DIA database, demonstrating that the HB cattle exhibited higher expression levels than the LB cattle, which aligns with the RNA-Seq trends (Figure 5C).

## 4. Discussion

A notable phenomenon has been extensively documented in the practice of cattle breeding: significant variations in the placental size among calves with differing birth weights. This observation holds considerable importance, as it suggests that calves with higher birth weights are likely to experience enhanced development into adulthood. Through a comparative correlation analysis of placental and neonatal phenotype data from Shandong black cattle, we found that the birth weight, leg circumference, and chest circumference of calves in the high-birth-weight (HB) group were significantly greater than those in the low-birth-weight (LB) group. Concurrently, the placental weight, length, and thickness of the HB group were also correspondingly elevated compared to those of the LB group. It can be inferred that both placental weight and thickness are positively correlated with newborn calf weight; specifically, a larger placenta correlates with increased newborn weight—a finding consistent with established knowledge within this field. Further analysis revealed that the HB group exhibited a higher degree of placental development within the uterus relative to the LB group. Calves in the HB cohort benefit from adequate nutritional supply and superior intrauterine development attributed to more fully developed placentae. This indicates enhanced intrauterine growth for the HB calves, potentially linked to their well-developed placentae. Additionally, substantial information such as daily weight gain—a critical indicator of fetal development—was included in other datasets, which also demonstrated differences between these two groups of calves. Although these differences were not as pronounced as those observed for birth weight, they nonetheless elucidate how placenta influences calf outcomes; notably, higher daily gains suggest improved fattening efficiency associated with better developmental trajectories postnatally. These observations corroborate previous research findings regarding placental function and calf development [45].

At the transcriptional level, our study elucidates that, during fetal development, the placenta of calves with a higher birth weight provides enhanced nutritional support to the fetus, thereby facilitating greater weight gain in these calves. This assertion is supported by three principal characteristics. First, placentae from high-birth-weight calves exhibit improved nutrient transport efficiency. This enhancement appears to be mediated by the upregulation of genes SLC2A1 and SLC2A11 within their placentae [46,47]; conversely, no significant differences in nutrient transport factors were observed in low-birth-weight calves. Further analysis reveals that significantly enriched differentially expressed genes (DEGs) are associated with the following four signaling pathways: transporter activity, active transmembrane transporter activity, membrane transport, and transmembrane transport—indicating distinct nutrient transport efficiencies and variations in placental development between both groups [48,49]. Second, high-birth-weight calf placentae demonstrate superior energy production capabilities. Notably, several genes involved in nutrient synthesis and transportation as well as energy metabolism—including ACSL1, MICALL2, PAG2, COL14A1, and ELOVL5—exhibit significant expression differences when comparing low- versus high-birth-weight groups [50,51,52,53,54]. These expression variations align with the increased nutritional demands of fetuses from the high-birth-weight group and correlate with other phenotypic metrics such as leg circumference and body height. Among these genes is ACSL1, which catalyzes the conversion of long-chain fatty acids into fatty acyl-CoA for subsequent oxidation or esterification reactions [55]. Additionally, ACSL1 accelerates initial steps in the fatty acid metabolism while promoting histone acetylation within adipocytes—a process influencing fat deposition—and serves as a crucial enzyme for lipid synthesis and catabolism [56,57]. The observed enrichment in the fatty acid metabolism and metabolic pathways further substantiates this perspective. Thirdly, placentae from higher-birth-weight calves display enhanced efficiency in fat synthesis—a conclusion drawn from the significant enrichment of differentially expressed genes related to the biosynthesis of unsaturated fatty acids, fatty acid elongation, and the Hippo signaling pathway. Notably, the downregulated differential genes are primarily involved in the regulation of multicellular organismal processes, cell migration, and angiogenesis. Previous studies have identified PAK1 and ITGA3 as two critical genes that promote both the proliferation and differentiation of stem cells while facilitating specific cell migration [58]; these genes are included among the differentially expressed proteins (DEPs) identified in this study. Cell proliferation-induced migration enhances the expression of extracellular matrix protein 1 (CTGF), thereby promoting extracellular matrix formation and accelerating placental development [59]. During placentation, the phosphorylation of PAK1 facilitates the repair of damaged chorionic villi, thus maintaining normal syncytiotrophoblast function [60]. The observed enrichment in the PI3K-Akt signaling pathway further supports this finding, indicating that placentae from the LB group prioritize responses to extracellular signals from placental cells, thereby enhancing the metabolism, proliferation, cell survival, growth, and angiogenesis within the placenta. In summary, at a transcriptional level, differences in calf birth weight can be attributed to enhanced nutrient provision by the placentae in the HB group—characterized by superior nutrient transport capabilities alongside increased energy metabolism and lipid synthesis—while those in the LB group appear more focused on cellular proliferation and angiogenic development.

At the protein level, our findings suggest that variations in calf birth weight among the Shandong black cows predominantly arise from the following two fundamental processes: the energy metabolism and lipid synthesis. Notably, placentae associated with higher birth weights demonstrate enhanced efficiency in both energy production and lipid synthesis, whereas those correlated with lower birth weights exhibited the superior regulation of essential biological processes such as cell migration, proliferation, differentiation, apoptosis, and signal transduction. A more comprehensive analysis indicates that upregulated proteins (ELOVL5, ELOVL7, TECR, HSD17B4, ACSL1, HACD2, and CBR4) are significantly enriched within the pathways of oxidative phosphorylation and metabolic pathways pertinent to amyotrophic lateral sclerosis. Prior research has established that ELOVL5 and ELOVL7 play a critical role in fatty acid elongation by adding two carbon units. This process involves specific enzymes (ACSL1) and cofactors (HACD2) catalyzing reactions wherein fatty acyl-CoA acts as a substrate alongside NADPH, serving as a reducing agent; this facilitates the stepwise addition of carbon atoms to the elongating fatty acid chain, ultimately yielding very-long-chain fatty acids [61]. Fatty acids are recognized not only for their role in providing energy to cells but also for their capacity to synthesize various lipids, including triglycerides and phospholipids [62]. Additionally, they can concentrate and transport essential long-chain unsaturated fatty acids to the fetus through several mechanisms, such as selective uptake by the chorionic membrane, intracellular metabolic transfer, and targeted delivery to fetal circulation [63]. While our findings substantiate that a high-birth-weight placenta enhances the energy efficiency for fetal development and accelerates the lipid synthesis, influencing birth weight, it is imperative to recognize that such placentae demonstrate diminished immunity against a range of diseases, including systemic lupus erythematosus and bacterial dysentery. Furthermore, it is important to note that proteins with elevated expression in the LB group placenta are significantly enriched in critical biological processes such as cell migration, proliferation, differentiation, apoptosis, and signal transduction. The downregulated proteins TNC, CNN3, HRG, and SPAG9 are again significantly enriched in the Gene Ontology (GO) term associated with the regulation of the cell migration pathway, further reinforcing the importance of GO term enrichment. Notably, the placenta in the LB group conferred enhanced disease immunity during fetal development compared to that in the HB group. The downregulated proteins SCIN, Igh-1a, and IGHG2 demonstrate significant enrichment within the Fc epsilon RI signaling pathway. The Fc ε RI complex is formed through interactions between the Fc region of antigen-specific immunoglobulin E (IgE) and high-affinity receptors on cell surfaces [64]. This complex regulates mast cell and basophil activation while facilitating IgE-mediated antigen presentation [65]. Upon activation, mast cells release stored granules containing proteoglycans (notably heparin) and biogenic amines (particularly histamine) [66]. Furthermore, Fc ε RI plays a pivotal role in initiating and sustaining allergic responses while establishing a physiological barrier during parasitic infections [67]. Lastly, the downregulated proteins COL6A1, TNC, CD36, CD81, Igh-1a, and IGHG are significantly enriched in both the ECM–receptor interaction and B cell receptor signaling pathways—further corroborating our hypothesis.

The integration of proteomics and transcriptomics elucidated the complementary and synergistic functions of genes and proteins. We identified 1036 upregulated genes, 458 downregulated genes, 217 upregulated proteins, and 77 downregulated proteins. Notably, nearly all proteins identified in the proteomic analysis were represented by their corresponding genes detected in the transcriptomic analysis, underscoring the efficacy of this study in analyzing core genes associated with fetal calf development. A higher number of upregulated DEPs was observed compared to those in the LB group, while fewer were downregulated. Importantly, our results indicate that the quantity of DEPs is significantly lower than that of the DEGs, with a correspondingly reduced number of detected proteins. This discrepancy may be attributed to their status following transcription, translation, post-translational modifications, and other complex processes [48,49]. Post-transcriptional modifications can alter protein structures such that protein expression does not always correlate with gene expression; this aspect warrants further investigation. Furthermore, we identified 53 genes and proteins exhibiting significant co-expression across both transcriptomic and proteomic analyses: among these are 40 co-upregulated proteins, 8 co-downregulated proteins, and 5 showing upregulation at the protein level but downregulation at the mRNA level. This observation is unsurprising given that the number of upregulated genes substantially exceeds that of the downregulated ones—potentially accounting for variations in the calf birth weight. Through the enrichment analysis, we determined that the majority of upregulated DEGs/DEPs were significantly enriched in critical biological processes, including the very-long-chain fatty acid metabolic process, lipid metabolic process, and lipid biosynthetic process. These processes encompass the following eight upregulated proteins: ELOVL5, ELOVL7, ABCD3, HACD2, RDH11, PIP4K2C, ACSL1, and CYP11A1. Notably, ACSL1, ELOVL5, HACD2, and ELOVL7 are involved in the fatty acid metabolism pathway, which provides enhanced energy for the growth and development of fetal calves. Additionally, ELOVL5, HACD2, and ELOVL7 are particularly enriched in fatty acid elongation. The regulatory mechanisms governing these proteins will not be elaborated upon here, as they have been previously detailed in the protein data. Importantly, we also observed that the cholesterol biosynthesis and metabolism were elevated in the placenta of the HB group. The co-expression of differentially expressed upregulated proteins (CYP11A1 and CYP17A1), which are significantly enriched within ovarian steroidogenesis pathways as well as steroid hormone biosynthesis and cortisol synthesis/secretion pathways, further substantiates this finding. CYP11A1 functions as a cholesterol side-chain cleavage enzyme integral to steroid hormone synthesis by catalyzing the conversion of cholesterol to pregnenolone [68]. Furthermore, the disruption of CYP11A1 gene expression can induce alterations in local microenvironment conditions along with inflammatory cytokine release from stromal cells; this may indirectly impair neural stem cell function, thereby potentially adversely affecting fetal neural development [69]. Moreover, the low methylation level of the CYP11A1 gene within the placenta from the HB group disrupts normal steroid hormone synthesis pathways, leading to restricted fetal development and an increased risk for pre-eclampsia [70]. In summary, our findings at the protein level indicate that the observed increase in calf birth weight within the HB group can be attributed to the following three primary pathways: the enhanced efficiency of energy production and lipid synthesis in the placenta, along with the cholesterol biosynthesis and metabolism.

Based on the positional and enrichment analyses of the PPI network, several proteins were identified, including ELOVL5, ELOVL7, and ACSL1. These proteins occupy central positions within the PPI network as key nodes that interact with other differentially expressed proteins (DEPs), demonstrating robust interactions and serving as core components. Integrating all experimental findings, we selected five differential genes/proteins—ELOVL5, ELOVL7, ACSL1, CYP11A1, and CYP17A1—as critical regulatory factors influencing calf birth weight. These proteins modulate the fatty acid metabolism, lipid synthesis, and cholesterol levels to impact fetal development. It is well established that various factors can influence normal placental nutrient transport; these include blood flow dynamics, placental size and morphology, as well as the abundance of transport proteins [71,72,73]. Fatty acids and cholesterol are essential nutrients for fetal growth and development [74]. In maternal circulation, free fatty acids exist either in their unbound form or as components of lipoproteins [75]. Fatty acids can be released from lipoproteins by fatty acid hydrolases (such as ELOVL5, ELOVL7, and ACSL1) located on the surface of trophoblast cells before being internalized through various transporter proteins. Cholesterol is similarly incorporated into lipoproteins and absorbed by the placenta via receptor-dependent or independent mechanisms within maternal circulation [76,77]. Furthermore, cholesterol can be synthesized by distinct ATP-dependent hydroxylases (CYP11A1 and CYP17A1) prior to its transport to either maternal or fetal circulation through specific transport proteins [68,78]. Collectively, these findings indicate that ELOVL5, ELOVL7, ACSL1, CYP11A1, and CYP17A1 may regulate nutrient accumulation to enhance placental function and fetal development, thereby influencing calf birth weight. Finally, 16 key candidate genes, including *ACSL1*, were selected for q-PCR validation. The results indicated that the real-time quantitative fluorescence data were consistent with the sequencing outcomes, reflecting a high degree of accuracy in the findings. These relevant candidate genes warrant further validation at the cellular level.

## 5. Conclusions

In this study, we propose that ELOVL5, ELOVL7, ACSL1, CYP11A1, and CYP17A1 function as protein markers that regulate calf birth weight through the modulation of the fatty acid metabolism, lipid synthesis, and cholesterol levels—ultimately impacting the birth weight of calves. The functional analysis of these differentially expressed genes/proteins deepens our understanding of essential biological processes and identifies potential biomarkers. These findings provide a substantial theoretical framework for the development of fetal calves.

## Data Availability

The mass spectrometry proteomics data have been deposited to the Proteomics Identifications Database (https://www.ebi.ac.uk/pride/archive, accessed on 3 July 2023) with the dataset identifier PXD043483. The username and password are reviewer_pxd043483@ebi.ac.uk and dWqEQxbO. The transcriptome data have been deposited to the NCBI (PRJNA989367).

## Supplementary Materials

The following supporting information can be downloaded at: https://www.mdpi.com/article/10.3390/ani14182751/s1, Figure S1: The distribution of peptide numbers and annotated protein numbers in different databases; Table S1: Primer pairs applied for qRT-PCR; Table S2: Sequencing statistics for each replicate.
