# Supplementary material for: Combining Transcriptomics and Proteomics to Screen Candidate Genes Related to Bovine Birth Weight
Source: Animals (Basel). 2024 Sep 23;14(18):2751. doi: 10.3390/ani14182751 (PMC11429316; doi:10.3390/ani14182751)
Supplement: Supplementary file 1 [file animals-14-02751-s001.zip › animals-3137660-supplementary.pdf]

Table S1. Primer pairs applied for qRT-PCR.

Gene name	Primer sequences(5'-3')	Annealing temperature(℃)	Product length(bp)
PAK1	F:GACATTTGGTCCTTGGGC<br>R:TTGTTCTTGGTTGCTTCC	51	296
ITGA3	F:GCGGAAGGACTGGGATT<br>R:CTGGCTCAGCAAAAAGA	51	175
GNAI1	F:AATGTTTGATGTGGGA<br>R:CGTGAAGTGGGTGTAT	45	382
PDLIM1	F:GTGACAGAGGAAGGGAA<br>R:GCAAAACCAGGAATGAA	51	379
PIP4K2C	F:GAAAGTGAAGGCAGTG<br>R:GTGGTAGTTGGACAGG	45	114
IQGAP2	F:AACGCTAAGAAACCCAAACG<br>R:ATACATCGCAGCAGCAAAAG	53	328
TMSB4X	F:GACAAACCCGATATGGC<br>R:CCTGCTTGCTTCTCCTG	48	119
PRKCI	F:GAACAGGCATCCAATCATCC<br>R:TCCTTCAGAGTCCAGCAACA	51	252
ACSL1	F:CCAGAGGGGCTTACAC<br>R:CTTCAGTTCCGAGGGT	51	266
ELOVL5	F:CCCCACTTTGGTCTGTTC<br>R:TCCCATACTCCCGTCACT	51	168
COL14A1	F:CCTTCAGTCTCAAGCCATGGTG<br>R:CGAGTGGCTGGGAATCTGGTTA	60	262
SERPINH1	F:TCTGGATGGGCAAGATGCAGAA<br>R:CTGTAAGTCGTGGGTCACCTCC	59.5	480
CD81	F:ACCTGCCTGGTGATCCTCTTTG<br>R:GCTTCACATCCTTGGCGATCTG	60	144
SLC23A2	F:ATGCCATGTGTGTGGGGTATGA<br>R:AACAGGGGTAACCTGCATCCAA	59.5	124
TAGLN	F:AATGGCGTGATTCTGAGCAAGC<br>R:TGCTCCATCTGCTTGAAGACCA	59	172
S100A13	F:GTTGCCTCACCTGCTCAAGGAT<br>R:TCTCCAGGGCCTTCTCTTTCCT	60	168

Table S2. Sequencing statistics for each replicate. GC: the percentage of G and C bases in the clean data with respect to the total bases; ≥ Q20%/≥Q30%: percentage of bases in the clean data above the Q20/Q30 threshold; Mapped Reads: successfully mapped paired-end reads.

Group	Sample ID	Clean reads	GC(%)	⩾Q20(%)	⩾Q30(%)	Mapped Reads
	LW1	40896120	2928446761（48.02%）	5891857314(96.61%)	5560566739 (91.18%)	285118
L	LW2	44090358	3219267208（48.88%）	6373795371(96.77%)	6032781788 (91.59%)	1078872
	LW3	39981692	2914559503（48.79%）	5732259738(95.97%)	5364762265 (89.81%)	286774

	HW1	41314794	2970527282（48.10%）	5934462590(96.08%)	5561329403 (90.04%)	592122
H	HW2	39669232	2719049218（45.98%）	5716080046(96.66%)	5392739623 (91.19%)	654458
	HW2	41652334	2969106092（47.74%）	6010604034(96.63%)	5669578197 (91.15%)	610786

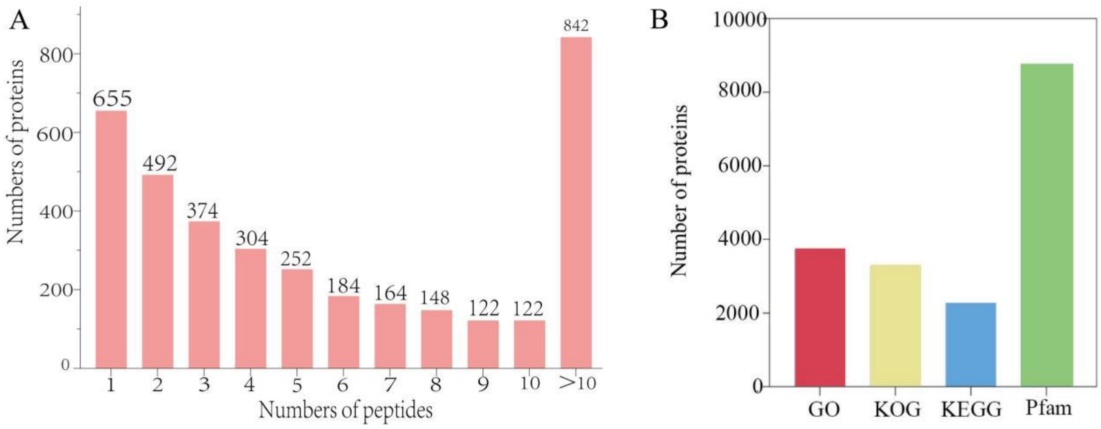

Figure S1. The distribution of peptide numbers and annotated protein numbers in different databases. (A) Distribution map of peptide number. (B)Protein database distribution.
